# A reconfigurable graphene patch antenna inverse design at terahertz frequencies

**DOI:** 10.1038/s41598-023-35036-4

**Published:** 2023-05-24

**Authors:** Mohammad Mashayekhi, Pooria Kabiri, Amir Saman Nooramin, Mohammad Soleimani

**Affiliations:** School of Electrical Engineering, Iran University of Science and Technology, Tehran, 1684613114 Iran

**Keywords:** Electronic properties and devices, Terahertz optics, Electrical and electronic engineering

## Abstract

This article investigates the inverse design of a reconfigurable multi-band patch antenna based on graphene for terahertz applications to operate frequency range (2–5THz). In the first step, this article evaluates the dependence of the antenna radiation characteristics on its geometric parameters and the graphene properties. The simulation results show that it is possible to achieve up to 8.8 dB gain, 13 frequency bands, and 360$^\circ$ beam steering. Then and due to the complexity of the design of graphene antenna, a deep neural network (DNN) is used to predict the antenna parameters by given inputs like desired realized gain, main lobe direction, half power beam width, and return loss in each resonance frequency. The trained DNN model predicts almost with 93% accuracy and 3% mean square error in the shortest time. Then, this network was used to design five-band and three-band antennas, and it has been shown that the desired antenna parameters are achieved with negligible errors. Therefore, the proposed antenna finds many potential applications in the THz frequency band.

## Introduction

Nowadays, the terahertz band is used in wireless telecommunications^1^, hyperthermia treatment of breast cancer^2^, biomedical imaging, security screening, and material identification^3^ due to its remarkable properties. In wireless communication, the need for multi-band antennas has increased due to a reduction in the number of antennas, a reduction in the complexity and cost of the system, and providing the possibility of integration with other circuits of the structure^4,5^.

On the other hand, the use of graphene has been very impressive in recent years in the field of Nano-electronic and THz devices due to its high conductivity and the changeability of the conductivity by tuning the bias voltage. The use of graphene in THz imaging^6^, patch antennas^7–9^ ultra-broadband absorbers^10^, and photoconductive antennas^11^ has been reported. In^12^, a dual-band antenna with an average gain of 2.45 dB is designed by creating two circle strips on the graphene. In^13^, a three-band frequency reconfigurable antenna has been proposed for a slotted patch graphene antenna. In^14^, a three-band antenna is implemented with a series feed circle graphene patch with a gain close to 10 dB. In^15^, a four-band antenna has been reported for a four L-shaped stub graphene patch antenna. Generally, for graphene antennas, it is possible to change the number of operating frequency bands by the variation of graphene chemical potential, similar to^16,17^ in which a four-band and three-band graphene antenna has been designed with the gain of 2.58 dB and 9.51 dB, respectively.

Making less computational time of resources with an acceptable result is of substantial importance in electromagnetic applications. In this regard, the machine learning approach has recently demonstrated outstanding performance compared to the computational and iterative methods in dealing with electromagnetic problems. Deep learning (DL) is a subset of machine learning (ML) with more robust computing capabilities, which is based on neural networks (NNs) and can learn the nexus between inputs and outputs. After learning, the designed model based on trained data can show a reasonable prediction as outputs for various given inputs in a fraction of a second. By taking advantage of this, DL was a suitable technique for inverse scattering problems^18,19^, design of metasurfaces^20–22^ and metamaterials^23–25^, design of photonic structures^26,27^,beamforming^28,29^, design of antennas^30–32^.

The deep neural network (DNN) architecture has been evolving over the years with new techniques and advancements. We can mention some recent techniques that applied in electromagnetic fields^23,27,33^. Usually, these DNN architectures include multiple networks to handle a specific part of the problem and have high structural complexity. Some of these techniques are used to solve the one-to-many issue. The one-to-many issue is a challenging problem in machine learning, which refers to a situation in which a single input is associated with multiple outputs. A tandem architecture is presented in^34^ by bringing together forward modeling and inverse design. The approach demonstrated in this model overcomes the one-to-many issue better than the inverse design model for the designing of nanophotonic structures. Then to overcome the limited generalization ability of^23^, a probabilistic graphic model introduced as an all-inclusive explanation for metamaterial design.

We have classified using the neural networks in antenna design into three approaches. First, NNs and ML enhance some radiation properties by optimizing the antenna parameters and can not control antenna radiation patterns in real-time^35^. Second, by giving the antenna dimensions as input, the antenna radiation will be estimated as output in real-time so DL and ML can speed up the antenna simulation directly^30,31^. And third, the most widely used method is the inverse design of the antenna using DL. The required radiation pattern characteristics are provided as input, and the DNN’s output estimates the antenna parameters. In this case, depending on the circumstances, the antenna parameters may be fully adjustable or non-adjustable^28,32^. Although in^29^ a $VO_2$ is used as a reconfigurable component in the antenna, the proposed DNN outputs geometrical antenna parameters.

In this article, an inverse design of reconfigurable graphene circular patch antenna at THz frequencies is proposed and surveyed to the realization of an intelligent antenna for 6G wireless communication. Also, for the first time we apply a chemical potential of graphene as a reconfigurable component in the output of DNN to control the radiation properties in real-time. The antenna parameters are divided into two groups, graphene and antenna parameters. After the analysis, we generated a data set and filtered it with two specific conditions. Then, a DNN model is presented, that can accurately predict the values of the graphene properties and antenna parameters for the desired S-parameters and radiation pattern of the antenna.

For this, in Sect. “Deep learning”, the design and simulation of the antenna have been discussed. Then, the used deep learning method will be explained and the achieved results have been examined in Sect. “Evaluation of deep neural network”. Finally, some conclusions are remarked.

## Antenna design and simulation

**Figure 1:**
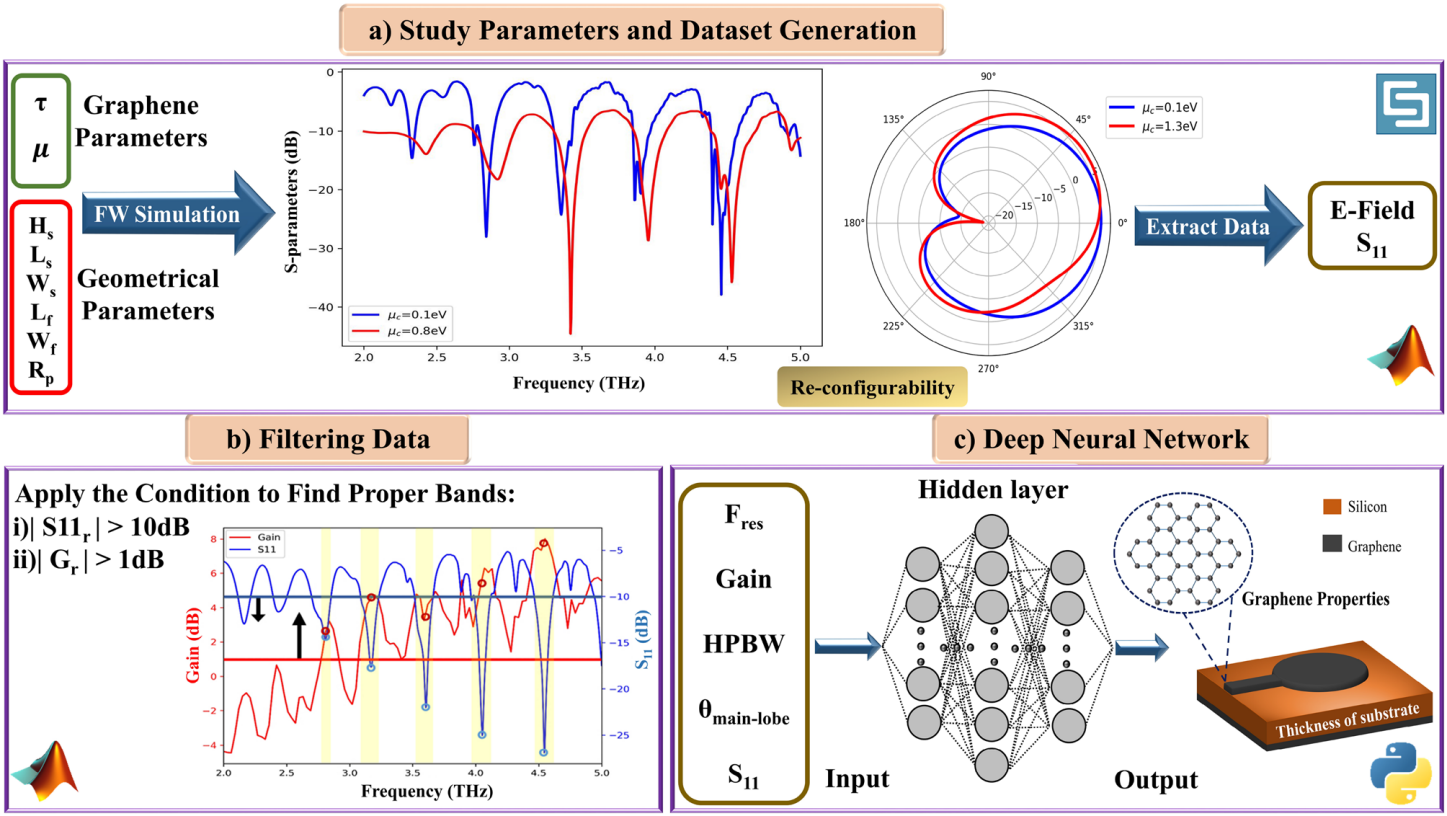
Flow chart of the main steps in the inverse design of graphene patch antenna.

The flow chart of the activity steps is plotted in Fig. 1. At first, the behavior and dependency of the antenna characteristics on its parameters are investigated by taking into account the antenna parameters are divided into two groups, graphene parameters and geometrical parameters. Then, the data needed for training the DNN model is generated by sweeping the graphene and geometrical parameters in antenna simulations (see Fig. 1a). Secondly, two filters are applied to extract features from S-parameters and E-field patterns, in order to gather proper data for use in the training data of the DNN model (see Fig. 1b). Finally, we present a DNN model, which can accurately estimate the values of the thickness of the substrate, $\tau$, and $\mu _c$ for desired inputs. In this work, Desired inputs of DNN comprise resonance frequencies, realized gain, null level, main lobe direction, and half-power beam width (see Fig. 1c). Based on the mentioned procedure, each section will be explained below.

### Antenna structure definition

**Figure 2:**
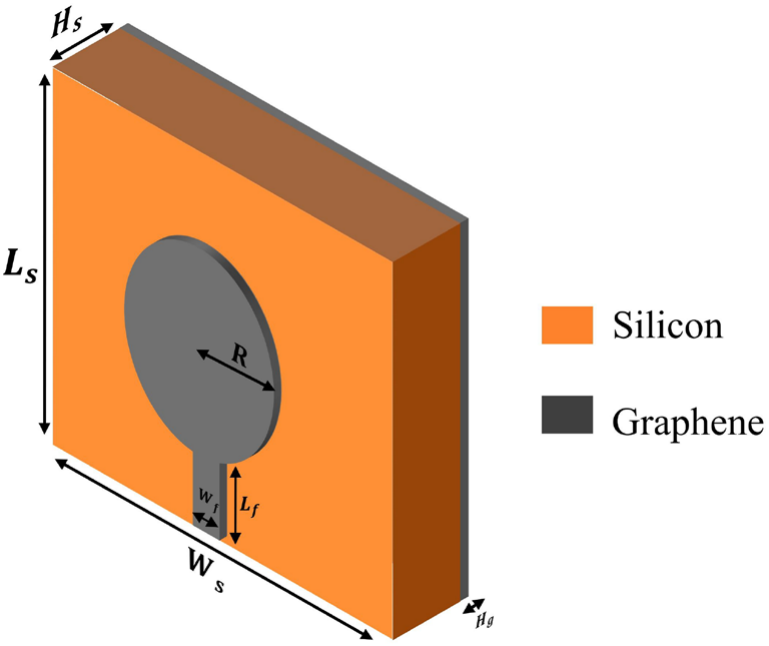
The structure of the patch antenna.

The structure of the antenna along with its parameters is plotted in Fig. 2. As shown in this figure, the structure is comprised of three layers. At the top and bottom, a graphene layer with thickness $H_g$ = 0.08mm and temperature $T_k$ = 273K is deposited. For the middle layer, a silicon layer by the relative permeability $\epsilon _r$ = 11.9 and conductivity $\sigma$ =$(25*10^{-5})$ is used. The other characteristics and dimensions of layers are given in Table 1. It is worth mentioning that during the simulations, a 50-ohm port impedance is assumed for the excitation lumped port, and the antenna parameters are determined such that input matching is achieved.

**Table 1:** Range of initial values of the antenna parameters.

Parameters	Range
$\mu _c$	[0–2] *eV*
$\tau$	[0.1–1] *ps*
$H_s$	[10–30] $\mu$ $m$
$W_s$ and $L_s$	[50–90] $\mu$ $m$
$R_p$	[15–25] $\mu$ $m$
$H_g$	[0.01–0.1] $\mu$ $m$
$W_f$	[4–12] $\mu$ $m$
$L_f$	[8–20] $\mu$ $m$

**Figure 3:**
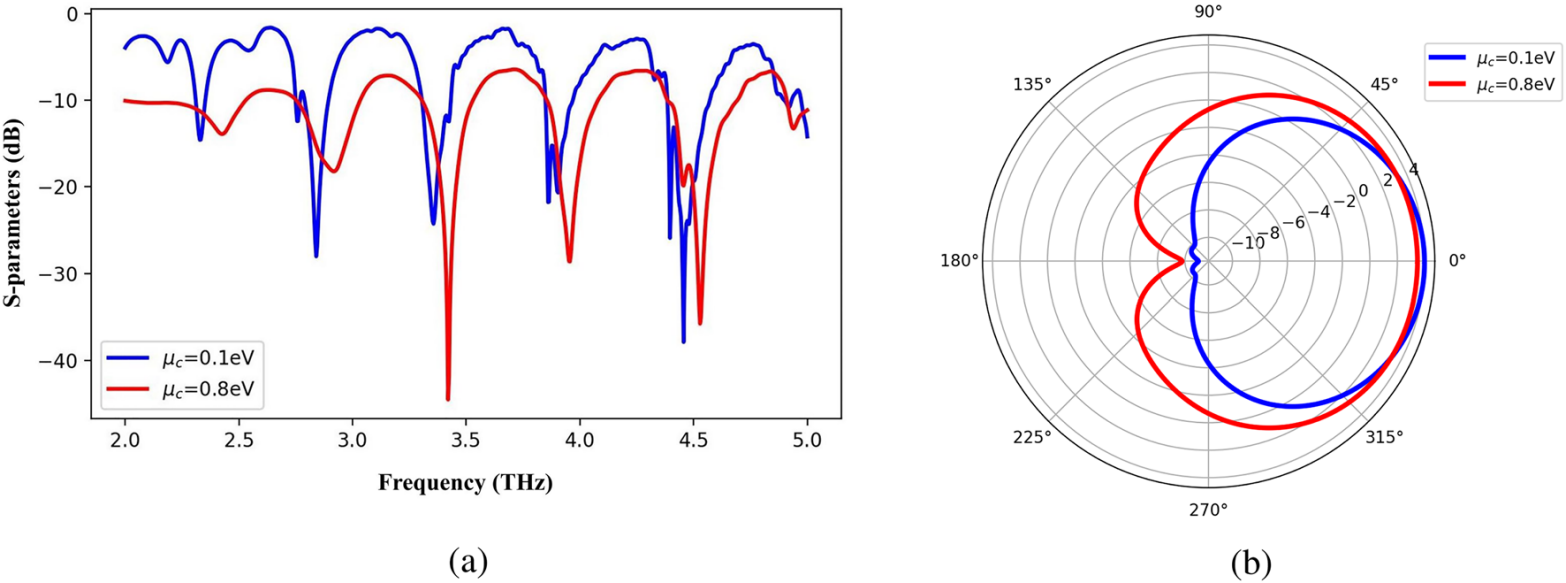
Changes in the return loss of the antenna due to variation of chemical potential (**a**) while the radiation pattern remains constant at the 2.34 THz frequency (**b**).

**Figure 4:**
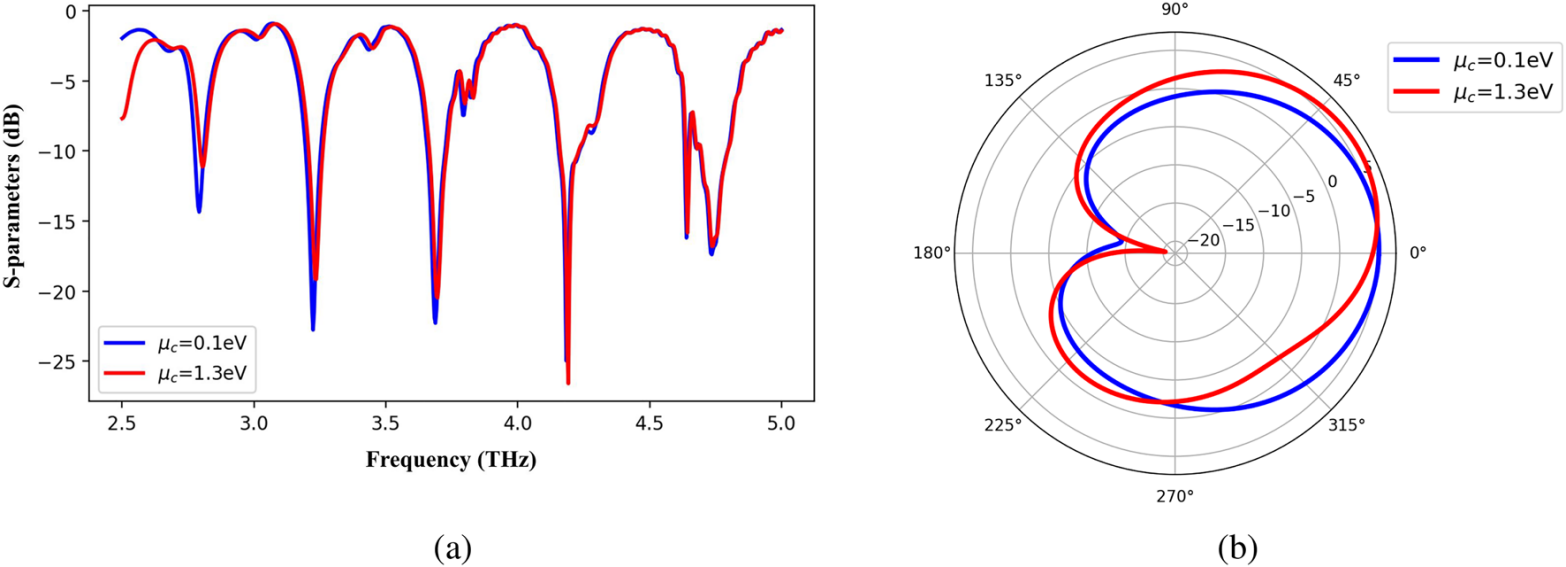
Relatively constant return loss (**a**) and 30-degree deviation of the beam direction due to changes in the chemical potential at the 3.21 THz frequency (**b**).

Due to the use of graphene in the patch and ground of the structure, a reconfigurable radiation pattern or a reconfigurable operating bandwidth is the inherent property of the structure. As shown in Fig. 3 , if the graphene chemical potential changes from 0.1 *eV* to 0.8 *eV*, the operation frequencies of the antenna have been shifted while the radiation pattern at the 2.34 THz frequency remains relatively constant. In this simulation, $H_s$ = 30 $\mu$ $m$, relaxation time = 0.1 *ps*, $W_f$ = 12 $\mu$ $m$, and $R_p$ = 18 $\mu$ $m$. Similarly, it is possible to change the beam direction by making changes in chemical potential as shown in Fig. 4 . It is obvious in this figure; for the changes of chemical potential from 0.1 *eV* to 1.3 *eV*, the return loss of the antenna remains relatively constant while the beam direction has been tilted about 30 degrees at the 3.21 THz frequency. In this simulation, $H_s$ = 20 $\mu$ $m$, relaxation time = 1 *ps*, $W_f$ = 6 $\mu$ $m$, and $R_p$ = 18 $\mu$ $m$. Therefore, the proposed antenna can be used as a reconfigurable antenna.

### Antenna parameter study and the generation of data sets

In this section, the effect of antenna parameters on the radiation properties is studied and the results are categorized to learn the neural network. For this, full-wave simulations are done in CST Microwave Studio. The study parameters include chemical potential ($\mu _c$ = [0-7] *eV*), relaxation time ($\tau$ = [0.1-2] *ps*), substrate thickness ($H_s$ = [20,30] $\mu$ $m$), the width of the substrate ($W_s$ = [70,90] $\mu$ $m$), the patch radius ($R_p$ = [18,25] $\mu$ $m$), the feed length ($L_f$ = 17 $\mu$ $m$), and the graphene thickness ($H_g$ = 0.08 $\mu$ $m$) . Finally, the number of simulations is equal to 2880. It is worth mentioning that the simulations are done in the frequency range of [1–5] THz and S$_{11}$, radiation pattern in E-plane, and realized gain is extracted in 101 equally spaced frequency points in the mentioned bandwidth. Then, the number and directions of main beams are extracted from the realized gain data based on the condition G$_{realized}$ > 1dB. Furthermore, the number of frequency bands is determined on the condition that |S$_{11}$| > 10dB. In the next step, the dependency of the antenna characteristics on the parameter values will be investigated.

### Studying the dependence of antenna characteristics on the values of its parameters

**Figure 5:**
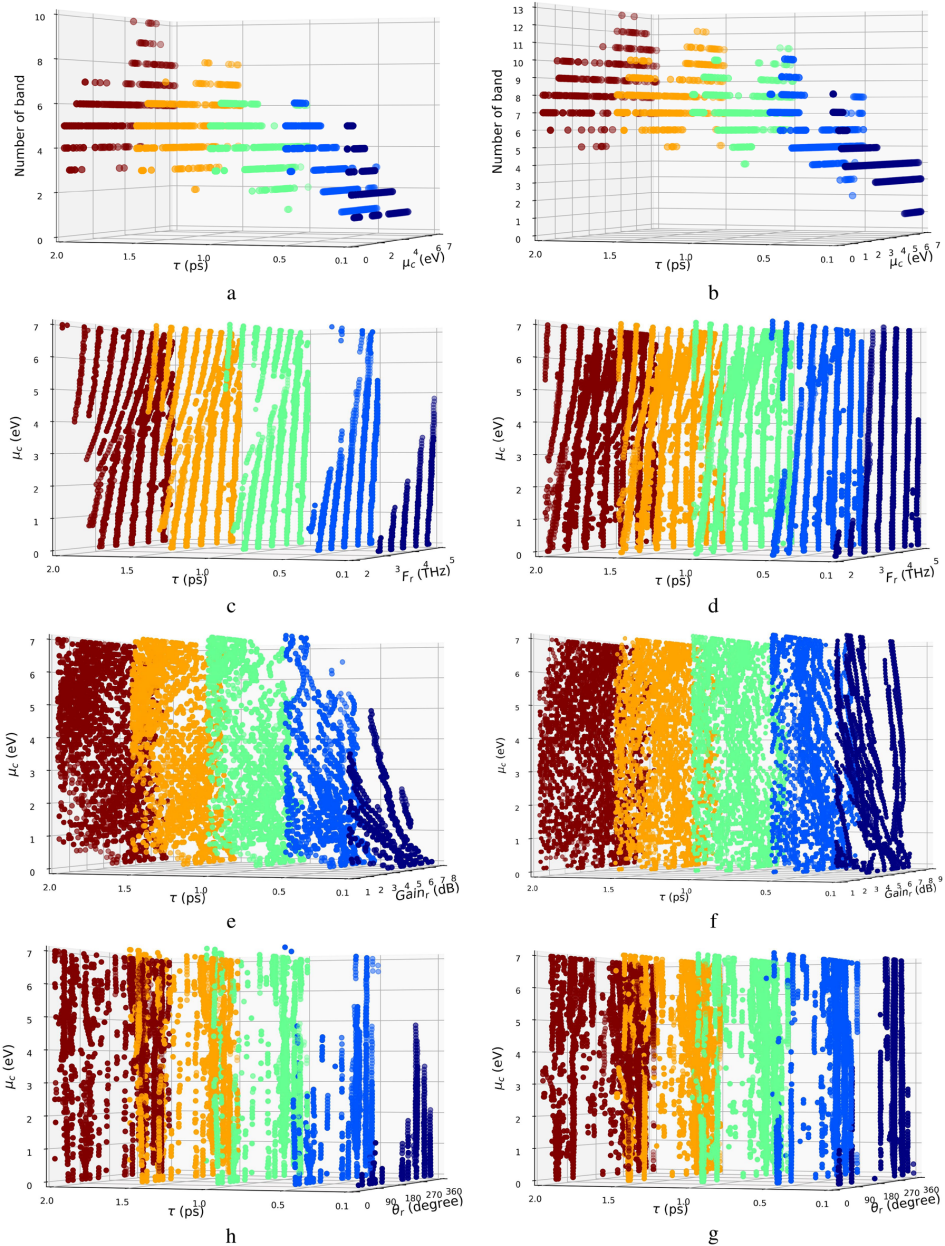
The study of the radiation properties of the antenna as a function of relaxation time ($\tau$), and chemical potential ($\mu _c$). The number of frequency bands (**a**,**b**), resonance frequency [THz] (**c**,**d**), gain [dB] (**e**,**f**), and main beam direction [degree] (**g**, **h**) are plotted as a function of relaxation time ($\tau$), and chemical potential ($\mu$c). in this simulation, (**a**, **c**, **e**, and **g**) are plotted for $H_s$ = 20 $\mu$ $m$ and (**b**, **d**, **f** and **h**) are plotted for $H_s$ = 30 $\mu$ $m$.

In Fig. 5a,b, the number of frequency bands is studied as a function of the relaxation time and the chemical potential for $H_s$ = 20 $\mu$ $m$ and $H_s$ = 30 $\mu$ $m$, respectively. As shown in this figure, higher chemical potential results into higher frequency bands. Also, more frequency band belongs to higher substrate thickness. Furthermore, changing the relaxation time will not affect the number of frequency bands for the zero chemical potential. It is worth mentioning that in Fig. 5, the color bar is devoted to relaxation time to make more clearance. Similarly, in Fig. 5c,d the center of the frequency bands which is named here as the resonance frequency, has been studied. As shown in this figure, multi-band operation is achieved for lower values of relaxation time chemical potential. Likewise, high values of chemical potential and relaxation time will result in higher frequency bands. Resonance frequencies in the range of [2–3] THz are achieved if $H_s$=30 $\mu$ $m$. In the same manner, Fig. 5e,f are devoted to studying gain. As shown in these figures, the values of gain are distributed in the range of [1–8.8]dB. Furthermore, higher gain values are achieved for thicker substrates and lower chemical potentials. In Fig. 5g,h, the angles of the main lobe directions are plotted. Based on the presented results in this figure, the distribution of the main lobe angle is wider for higher values of relaxation time, chemical potentials, and substrate thickness and can cover all the range of [0–360] degrees.

## Deep learning

**Figure 6:**
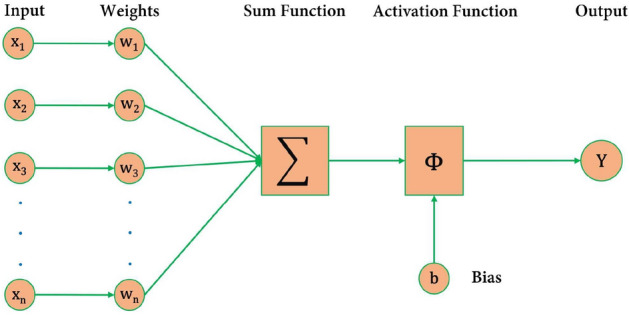
Schematic of neural network.

Artificial neural networks are machine learning techniques inspired by the human nervous system. The neural network consists of interconnected neurons, and by changing the weight of the interconnected neurons, that can learn the relationship between inputs and outputs by adjusting the weights of these connections, allowing them to generalize based on empirical knowledge. Figure 6 shows the schematic of a neural network. In this figure, *n* is the number of inputs and $x_i$ denotes the value of each input. Each input is associated with a specific weight, $w_i$, which is multiplied by the input and summed up. After summation, an activation function $\phi$ is applied to estimate the output, which can be biased with an initial value $b_i$. In equation (1), the output and input of the neural network are presented. Neural networks typically consist of an input layer, one or more hidden layers and an output layer, with each layer containning a specific number of neurons. Also, the weights of each neuron will change during a back propagation process to learn the input and output relationship pattern. In general, as the number of neurons and hidden layers increases, the ANN network becomes more DNN, which will cause the complexity of the model.

1 $$\begin{aligned} Y= \phi \left( \sum _{i=1}^{n} W_i X_i + b_i \right) \end{aligned}$$

In this research, the inputs of the deep neural network include the number of bands, the resonance frequencies of the bands, the beam direction, the half-power beam width, the gain, and the depth value of $S_{11}$ at each resonance frequency of the antenna. After the presented DNN has been learned, the required antenna parameters, such as the dimensions of the antenna and the properties of the required graphene, are estimated. As mentioned, the maximum number of bands achieved is 13, while the desired parameters total 5 in total. Therefore, each input vector is a row matrix of order 65, including the vector for each of the 13 frequency bands. In the proposed learning procedure, 60% of the data has been used for learning while the others are used in the test sequence.

The output of the neural network includes a third-order vector comprises the thickness of the substrate, relaxation time, and chemical potential. Since the goal of this research is to accurately estimate the continuous values, the ReLU activation function has been used in each network layer. It should be noted that the number of layers and neurons was optimized to achieve the best performance in the proposed network. In the proposed model, the batch size and learning rate are 512 and 0.001, respectively, and the  number of epochs is set to 5000. Also, the Adam optimization algorithm has been employed to determine the values of the weights in the model. Furthermore, the utility cost function MSE (Mean Squared Error) has been used to calculate the difference between the real value and the value estimated by the model, as given in equation (2), where  $y_i$ and $f_i$ represents the real and estimated values, respectively.

2 $$\begin{aligned} MSE=\frac{1}{N} \sum _{i=1}^{N} (f_i - y_i)^2 \end{aligned}$$

To achieve the best performance of the DNN, we conducted several tests on the combination of layers and their output shape value. Table 2 displays the optimal structure of the DNN for learning the relationship between inputs and outputs.

**Table 2:** The configuration of proposed DNN model.

Layers	Activation	Output shape	Parameters number
Dense	Relu	(None, 65)	4290
Dense	Relu	(None, 200)	13200
Dropout	–	(None, 200)	0
Dense	Relu	(None, 200)	40200
Dropout	–	(None, 200)	0
Dense	Relu	(None, 200)	40200
Dropout	–	(None, 200)	0
Dense	Relu	(None, 3)	603

**Figure 7:**
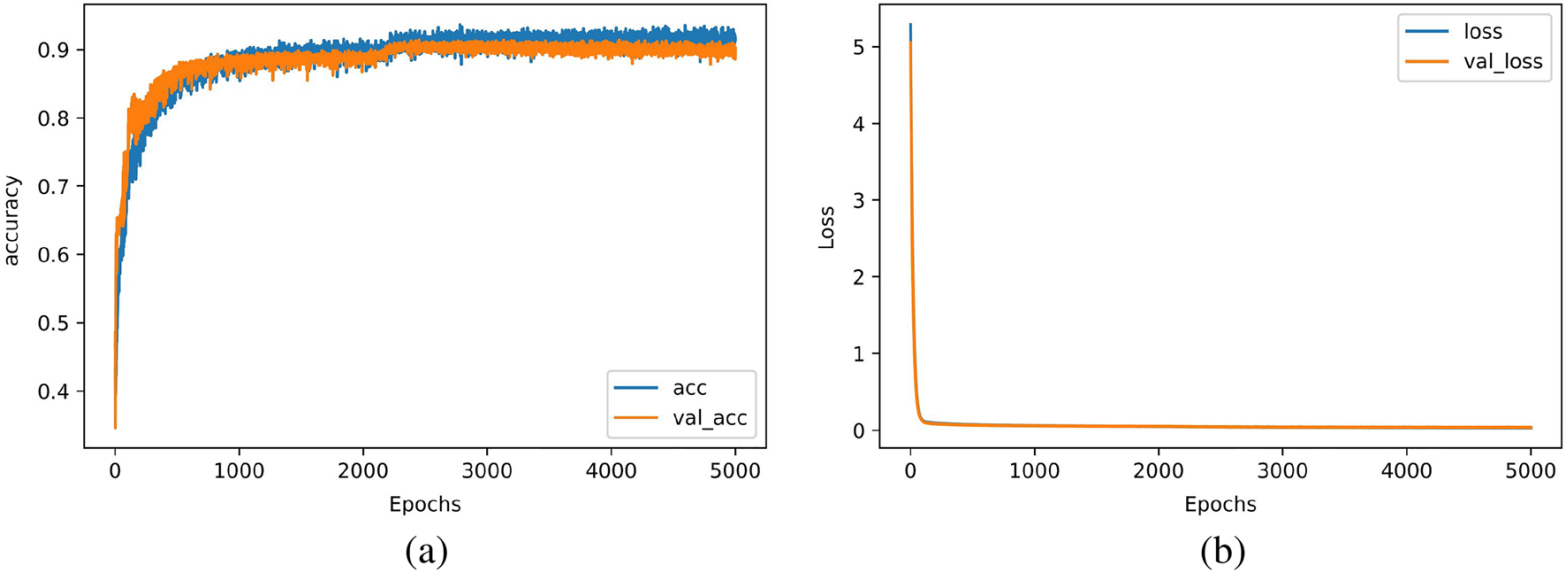
(**a**) accuracy and (**b**) loss of the proposed DNN.

The loss and accuracy diagrams of the proposed model have been sketched in Fig. 7a,b, respectively. As can be seen in this figure, the values of accuracy and loss achieve 91.5% and 0.03, respectively. Furthermore, it can be seen that the validation graph closely aligns with the training graph, indicating successful avoidance of overfitting in the model. In this procedure, the training time took about 262 seconds and the model can estimate the output in less than 0.05 seconds which was obtained on a system equipped with an Intel Core i7-10750H processor and 16GB RAM.

## Evaluation of deep neural network

**Figure 8:**
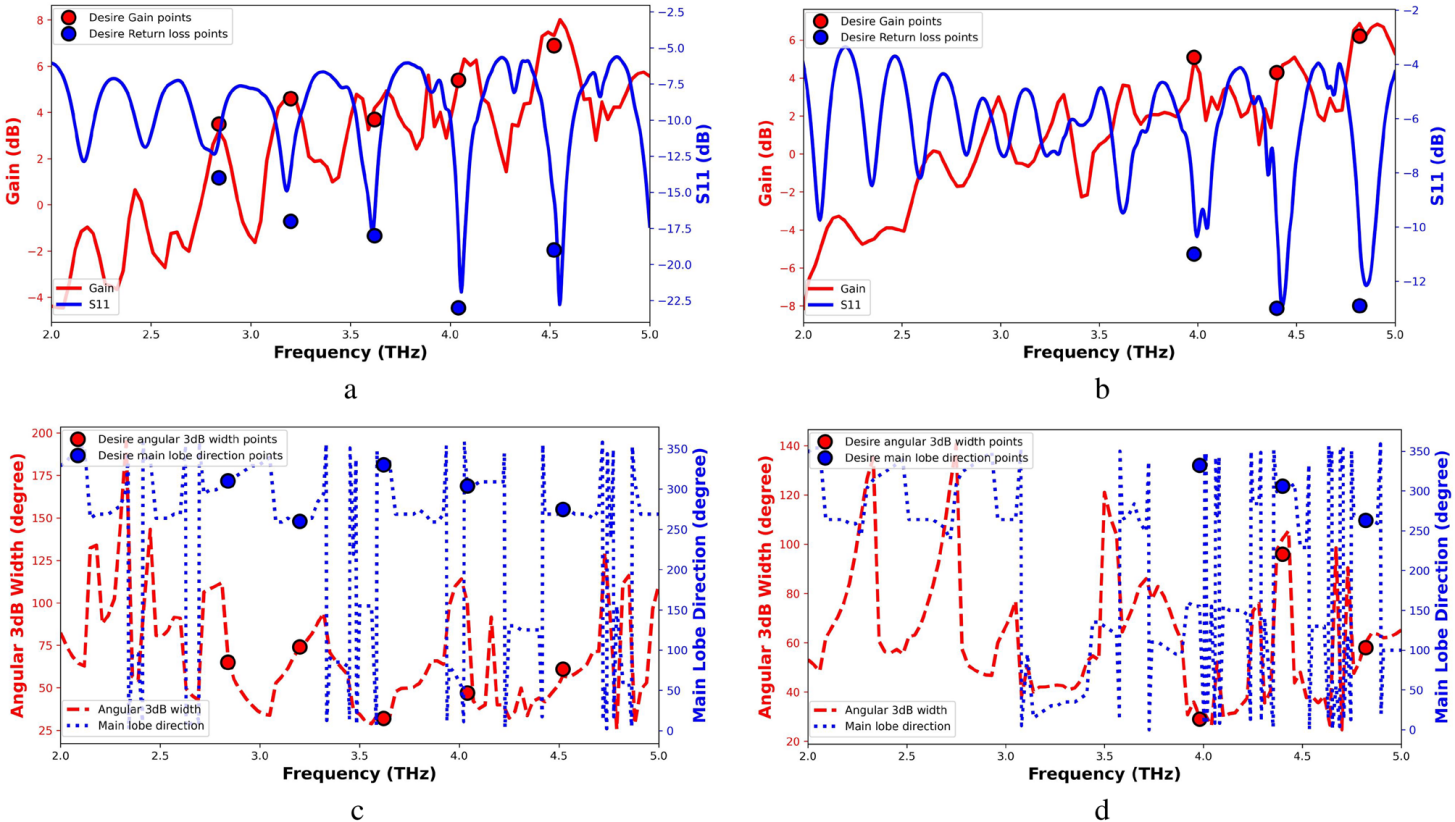
(**a**) and (**b**) simulation and expected values gain and $S_{11}$ for a five-band and three-band antenna, respectively. (**c**) and (**d**) simulation and expected values of half-power beam width and main lobe direction for five-band and three-band antenna, respectively. In this figure, expected values have been specified by colored circles.

For evaluation of the proposed model, we have provided two arbitrary samples whose specifications are given in Figure 8. For this, the desired values are fed to the proposed network and the antenna parameters are estimated. Subsequentlly, a full wave analysis was conducted to simulate the performance of the antenna. As shown in Fig. 8a,b, the gain and return loss of a five-band and a three-band antenna are plotted versus frequency, respectively. In these figures, the desired values are also shown using colored circles and are in good agreement with the simulation results. In the same way, the simulation results and the desired values of the half-power beam width and the main lobe direction for five-band and three-band antennas are plotted in Fig. 8c, d, respectively. The small differences between the simulated and expected values are also evident in these figures. The estimated values of the substrate thickness, chemical potential, and relaxation time are presented in Table 3. Furthermore, the MSE of the resonance frequencies, half-power beam width, gain and return loss are shown in table 3. According to these results, it can be claimed that negligble errors in achieving the desired antenna parameters are obtained through the estimation of antenna thickness and graphene properties.

**Table 3:** Estimated values and Mean Square Error (MSE) of predicted and real antenna properties. performance.

Examples	Estimated outputs			MSE between $f_{i}$ and$y_{i}$				
	$H_s$	$\tau$	$\mu _c$	$F_r$	$G_r$	$\theta _r$	$HP_r$	$Null_r$
Five-band	30 $\mu m$	0.50 ps	3.13 eV	$1.2e^{-5}$	$1.92e^{-3}$	$1.69e^{-5}$	$2.3e^{-4}$	$25e^{-4}$
Three-band	20 $\mu m$	0.94 ps	2.64 eV	$4e^{-5}$	$25.7e^{-3}$	$3.59e^{-5}$	$6e^{-4}$	$2e^{-4}$

## Discussion

In this article, a planar graphene antenna was investigated and its radiation characteristics for different substrate thicknesses and graphene characteristics including chemical potential and relaxation time have been extracted by full-wave FEM simulations. Then, these parameters were examined as the design inputs and it was shown that in the proposed structure, by choosing the appropriate values for the input, the number of bands and their resonance frequencies, antenna gain and main lobe directions can be set. The simulations have been performed in the [2–5] THz frequency band and it has been shown that the maximum gain of 8.8dB and up to 13 frequency bands can be achieved. Due to the complexity of the design and in the following, a deep neural network has been used to provide a design solution. This network is trained based on the categorized simulation results and the network parameters are determined in such a way that the most accurate matching between the estimated and simulated parameters has been achieved for minimum input data. In the end, it has been shown that the optimal radiation parameters can be estimated with an error of less than 3%. This feature can be used to design antennas in various applications. In the simulations, we encountered one-to-many issues. In fact, the analysis of the simulation results shows that for the antenna parameters of [$W_s$, $L_s$, $R_p$], relatively the same results for $S_{11}$ and E-fields may be generated. Furthermore, if all the structural antenna parameters are assumed as the outputs, errors would arise in estimating the input parameters. However, we considered this category of samples in order to maintain the number of neural network training dataset samples and improve the accuracy of the model, three parameters of [$\mu _c$ , $\tau$, $H_s$] are estimated as the output.

## Data Availability

The data that support the findings of this study are available from A.N. but restrictions apply to the availability of these data, which were used under license for the current study, and so are not publicly available. Data are however available from the authors upon reasonable request and with permission of A.N. (email: a_nooramin@iust.ac.ir).

## References

[1] Rappaport TS (2019). Wireless communications and applications above 100 GHz: Opportunities and challenges for 6g and beyond. IEEE Access.

[2] Mahmoud KR, Montaser AM (2022). Design of multi-resonance flexible antenna array applicator for breast cancer hyperthermia treatment. IEEE Access.

[3] Malhotra I, Jha KR, Singh G (2018). Terahertz antenna technology for imaging applications: A technical review. Int. J. Microw. Wirel. Technol..

[4] Mahabub A, Rahman MM, Al-Amin M, Rahman MS, Rana MM (2018). Design of a multiband patch antenna for 5g communication systems. Open J. Antennas Propag..

[5] Maci S, Biffi Gentili G (1997). Dual-frequency patch antennas. IEEE Antennas Propag. Mag..

[6] Hosseininejad SE (2019). Reprogrammable graphene-based metasurface mirror with adaptive focal point for thz imaging. Sci. Rep..

[7] Alibakhshikenari M (2021). High-isolation antenna array using SIW and realized with a graphene layer for sub-terahertz wireless applications. Sci. Rep..

[8] Khan MAK, Ullah MI, Kabir R, Alim MA (2020). High-performance graphene patch antenna with superstrate cover for terahertz band application. Plasmonics.

[9] Shamim SM, Das S, Hossain MA, Madhav BTP (2021). Investigations on graphene-based ultra-wideband (UWB) microstrip patch antennas for terahertz (THz) applications. Plasmonics.

[10] Liu L, Liu W, Song Z (2020). Ultra-broadband terahertz absorber based on a multilayer graphene metamaterial. J. Appl. Phys..

[11] Nissiyah GJ, Madhan MG (2019). Graphene-based photoconductive antenna structures for directional terahertz emission. Plasmonics.

[12] Nickpay MR, Danaie M, Shahzadi A (2022). Wideband rectangular double-ring nanoribbon graphene-based antenna for terahertz communications. IETE J. Res..

[13] Shalini M (2020). Performance predictions of slotted graphene patch antenna for multi-band operation in terahertz regime. Optik.

[14] Vijayalakshmi K, Selvi CS, Sapna B (2021). Novel tri-band series fed microstrip antenna array for THz MIMO communications. Opt. Quantum Electron..

[15] Bokhari BSM, Bhagyaveni MA, Rajkumar R (2021). On the use of graphene for quad-band THz microstrip antenna array with diversity reception for biomedical applications. Appl. Phys. A Mater. Sci. Process..

[16] Nissiyah GJ, Madhan MG (2021). Graphene based microstrip antenna for triple and quad band operation at terahertz frequencies. Optik.

[17] Kavitha S, Sairam KV, Singh A (2022). Graphene plasmonic nano-antenna for terahertz communication. SN Appl. Sci..

[18] Wei Z, Chen X (2019). Deep-learning schemes for full-wave nonlinear inverse scattering problems. IEEE Trans. Geosci. Remote Sens..

[19] Li Y (2020). Predicting scattering from complex nano-structures via deep learning. IEEE Access.

[20] Ma W (2022). Pushing the limits of functionality-multiplexing capability in metasurface design based on statistical machine learning. Adv. Mater..

[21] Ghorbani F (2021). Deep neural network-based automatic metasurface design with a wide frequency range. Sci. Rep..

[22] Zhu D, Liu Z, Raju L, Kim AS, Cai W (2021). Building multifunctional metasystems via algorithmic construction. ACS Nano.

[23] Ma W, Cheng F, Xu Y, Wen Q, Liu Y (2019). Probabilistic representation and inverse design of metamaterials based on a deep generative model with semi-supervised learning strategy. Adv. Mater..

[24] Raju L (2022). Maximized frequency doubling through the inverse design of nonlinear metamaterials. ACS Nano.

[25] Ma W, Cheng F, Liu Y (2018). Deep-learning-enabled on-demand design of chiral metamaterials. ACS Nano.

[26] Ma W (2021). Deep learning for the design of photonic structures. Nat. Photonics.

[27] So S, Rho J (2019). Designing nanophotonic structures using conditional deep convolutional generative adversarial networks. Nanophotonics.

[28] Tan YJ (2022). Self-adaptive deep reinforcement learning for THz beamforming with silicon metasurfaces in 6G communications. Opt. Express.

[29] Dao R-N (2023). The reverse design of a tunable terahertz metasurface antenna based on a deep neural network. Microw. Opt. Technol. Lett..

[30] Shi LP, Zhang QH, Zhang SH, Yi C, Liu GX (2021). Efficient graphene reconfigurable reflectarray antenna electromagnetic response prediction using deep learning. IEEE Access.

[31] Stankovic ZZ, Olcan DI, Doncov NS, Kolundzija BM (2022). Consensus deep neural networks for antenna design and optimization. IEEE Trans. Antennas Propag..

[32] Sharma K, Pandey GP (2021). Efficient modelling of compact microstrip antenna using machine learning. AEU - Int. J. Electron. Commun..

[33] Dong Q (2023). Plasmonic nanostructure characterized by deep-neural-network-assisted spectroscopy invited. Chin. Opt. Lett..

[34] Liu D, Tan Y, Khoram E, Yu Z (2018). Training deep neural networks for the inverse design of nanophotonic structures. ACS Photonics.

[35] Shi D, Lian C, Cui K, Chen Y, Liu X (2022). An intelligent antenna synthesis method based on machine learning. IEEE Trans. Antennas Propag..

